# Enhancing brain tumor detection: a novel CNN approach with advanced activation functions for accurate medical imaging analysis

**DOI:** 10.3389/fonc.2024.1437185

**Published:** 2024-09-20

**Authors:** Reham Kaifi

**Affiliations:** ^1^ Department of Radiological Sciences, College of Applied Medical Sciences, King Saud bin Abdulaziz University for Health Sciences, Jeddah, Saudi Arabia; ^2^ King Abdullah International Medical Research Center, Jeddah, Saudi Arabia; ^3^ Medical Imaging Department, Ministry of the National Guard—Health Affairs, Jeddah, Saudi Arabia

**Keywords:** brain tumors, Convolutional neural networks, detection, medical imaging, computer-aided diagnosis tools (CAD)

## Abstract

**Introduction:**

Brain tumors are characterized by abnormal cell growth within or around the brain, posing severe health risks often associated with high mortality rates. Various imaging techniques, including magnetic resonance imaging (MRI), are commonly employed to visualize the brain and identify malignant growths. Computer-aided diagnosis tools (CAD) utilizing Convolutional Neural Networks (CNNs) have proven effective in feature extraction and predictive analysis across diverse medical imaging modalities.

**Methods:**

This study explores a CNN trained and evaluated with nine activation functions, encompassing eight established ones from the literature and a modified version of the soft sign activation function.

**Results:**

The latter demonstrates notable efficacy in discriminating between four types of brain tumors in MR images, achieving an accuracy of 97.6%. The sensitivity for glioma is 93.7%; for meningioma, it is 97.4%; for cases with no tumor, it is 98.8%; and for pituitary tumors, it reaches 100%.

**Discussion:**

In this manuscript, we propose an advanced CNN architecture that integrates a newly developed activation function. Our extensive experimentation and analysis showcase the model's remarkable ability to precisely distinguish between different types of brain tumors within a substantial and diverse dataset. The findings from our study suggest that this model could serve as an invaluable supplementary tool for healthcare practitioners, including specialized medical professionals and resident physicians, in the accurate diagnosis of brain tumors.

## Introduction

The brain is an essential organ responsible for regulating the nervous systems in both humans and animals (1). Brain tumors are severe conditions characterized by abnormal cell proliferation within or near the brain, often resulting in high mortality rates (2). These tumors can be categorized as either primary or secondary based on their point of origin and as benign or malignant based on their characteristics. Primary tumors develop within the brain, while secondary tumors metastasize to the brain from elsewhere. Malignant tumors exhibit rapid growth and tend to infiltrate healthy cells in other body areas (3). The early detection and accurate diagnosis of brain tumors is crucial for effective treatment and improving patient outcomes.

Some prevalent forms of primary brain tumors consist of gliomas, meningiomas, pituitary adenomas, and schwannomas. Gliomas, mainly grades 3 and 4, are the most frequently occurring malignant tumors. Meningiomas and pituitary tumors are the prevailing benign tumors. Meningiomas arise from the membranes enveloping the spinal cord and brain, while pituitary tumors develop from the pituitary gland at the brain’s base (4, 5).

Imaging techniques, such as magnetic resonance imaging, are widely employed for visualizing the brain and identifying tumors. MRI utilizes a combination of a magnetic field and radio waves to generate detailed images of body tissues and organs, facilitating precise diagnosis and evaluation of tumor-related damage (6). In certain instances, tumor typing may require a biopsy. Improvements in computer technology have enhanced processing speed and memory capabilities. Many healthcare professionals acknowledge the necessity for creating reliable predictive models to confront their ongoing challenges. These models aim to recognize and categorize patterns, assisting medical experts in patient diagnosis and treatment strategies (7).

Deep Learning, a subset of machine learning, has become widely known for its ability to construct intricate structures or non-linear transformations through neural networks with multiple layers. This approach has shown significant promise in radiology and medical imaging by enhancing the precision and speed of tasks like image classification, segmentation, and anomaly detection (8).

Training deep learning models on extensive medical images allows them to extract essential features and patterns autonomously. This capability empowers them to recognize and categorize various medical conditions, such as brain tumors, lung diseases, and cardiovascular abnormalities (9).

Various medical imaging modalities, including MRI, CT, and PET scans, have benefited from successfully applying different types of deep learning models like convolutional neural networks. These CNNs are designed with convolutional layers for extracting features and densely connected layers for classification (10). Through extensive image data analysis and understanding of intricate patterns, CNNs have enhanced disease diagnosis and prognosis accuracy. A standard CNN typically comprises four layers: a convolutional layer, an activation function layer, a pooling layer, and a fully connected layer. These layers work together to process the input image, extract relevant features, and make predictions based on those features (11).

The activation function layer enables the network to capture non-linear correlations between input and output. Incorporating non-linearity into the network facilitates the representation of intricate patterns and connections within the data (12).

Transfer learning is commonly used to simplify the development of new models for similar tasks. It entails utilizing a pre-trained model trained on a large dataset as a basis for a specific task. This approach enables us to capitalize on the knowledge and features already captured by the pre-trained model, eliminating the necessity to train an entirely new model from the beginning. Numerous transfer learning models exist, including VGG, EfficientNet, ResNet, Inception, MobileNet, DenseNet, and others (13).

This work is applicable in the field of medical imaging, specifically for detecting and diagnosing brain tumors using advanced deep-learning models. The proposed method utilizes Convolutional Neural Networks (CNNs) with tailored activation functions to improve the accuracy and efficiency of brain tumor detection in MRI or CT scan images. The aim is to support radiologists, residents, and medical professionals by offering a more precise and automated tool for early diagnosis, which is essential for effective treatment planning. The proposed work introduces an innovative approach to brain tumor detection by developing and implementing a novel CNN architecture enhanced with advanced activation functions. This CNN model is specifically designed to analyze medical imaging data, such as MRI or CT scans, and accurately identify the presence of brain tumors. The primary innovation is the introduction of these advanced activation functions, which enhance the model’s capacity to recognize intricate patterns and subtle features within medical images. This approach can potentially enable more reliable and earlier detection of brain tumors, ultimately improving patient outcomes.

This study involved the development of a novel model utilizing CNN to categorize various forms of brain cancer using (MRI). We have made the following critical contributions to this study:

a. This study introduces an innovative CNN methodology for classifying four distinct types of brain tumors: glioma, meningioma, pituitary tumors, and no tumor.
b. We utilized various activation functions and conducted a comparative analysis between them.
c. After comparing them, we proposed a new type of activation function, which proved to be the most effective.
d. By attaining the best accuracy score on the Kaggle dataset, the study’s findings demonstrate that the suggested methodology performs better than current methods. Comparisons with previously used techniques and pre-trained models were also made to evaluate the approach’s prediction ability.

The remaining sections of this paper are structured as follows: Section 2 provides an overview of the related work, while Section 3 describes the proposed model for brain tumor classification. In Section 4, we present the experimental results. Finally, Section 5 concludes the paper.

## Related work

The intricate clinical significance of brain tumor classification using MR images makes this an important study field. Automation can be achieved using Deep Learning (DL) and Machine Learning (ML). The manual tumor segmentation and feature extraction processes of ML-based systems are their bottlenecks. DL-based methods, such as Convolutional Neural Networks (CNNs), are gaining ground on diagnosis challenges due to their superior performance in medical image processing. In this section, we focused on methods for brain tumor classification using deep learning. Researchers either utilized the same dataset in this study or created new CNN architectures and applied them to different datasets. Additionally, we included studies that employed transfer learning.

Elena McCain-Nino and her colleagues created a CRISP-DM model to identify brain cancers by adding a classification header to a ResNet-50 framework (14). They constructed a data generator to split pixels by 255 using a dataset of 3847 brain MRI images scaled to 256 × 256. The model demonstrated 92% accuracy and 94% precision throughout the test procedure. Using three distinct networks, including GoogleNet, Manish and Rajiv (15) used the idea of transfer learning to diagnose brain cancer images. 224 of the 696 MR-type images in the dataset are benign, while 472 are malignant. An accuracy of 96.65 is attained using the suggested framework.

Muhammad Altaf et al. proposed an approach to classification that uses pre-trained Google Net and deep transfer learning (16). This work examines a three-class method to differentiate between three prevalent brain cancer types: pituitary tumors, meningiomas, and gliomas. They used 930 images of pituitary tumors, 708 images of meningiomas, and 1426 MRI images of brains affected by gliomas. The accuracy was 91% when they used updated GoogleNet to power independently softmax classifiers. It is important to note that a hybrid approach that combines deep learning and machine learning was used in this study to improve accuracy. Accuracy increased to 95% after machine learning techniques were applied.

The seven recent CNNs for categorizing meningioma, glioma, and pituitary-type cancers were assessed by Anaya-Isaza et al. (17). The outcomes show that neural network detection and classification methods are quite good. First, the InceptionResNetV2 network outperformed the other networks with accuracy levels as high as 97%—next, a new network design known as the cross-transformer was added to the experiment. The FLAIR sequence was more effective in detecting brain tumors, with an essential level below 0.03 in six out of eight networks. Furthermore, it was demonstrated that the cross-transformer obtained accuracy values that were nearly 90%.

Ravinder et al. (18) proposed a CNN-based model to predict brain tumor types (Meningioma, Pituitary, Glioma, or No tumor) using non-Euclidean distances in image data. The model achieved an accuracy of 95.01%, with Net-2 with Graph input-based CNN and Gaussian Adjacency matrix achieving the highest accuracy. This model is considered a vital alternative for detecting brain tumors in suspected patients.

The study in (1) investigated the CNN structure and its ability to classify data, and the low number of layers revealed shortcomings. The effect of the VGG16Net, in addition to DenseNet models, on success rates was examined. The approach was not applied since transfer learning had no discernible impact on the success rate in the health industry. Positive outcomes in thick layers were shown by DenseNet analysis; however, not to the anticipated extent. The training step was finished in person using CNN architecture. It was possible to locate a dataset with approximately 7,000 photos split between 80% training and 20% testing phases. The model’s success rate was 94–97% without using transfer learning techniques.

This study created a CNN In (19) to identify brain cancers from MRI data. The network was trained using a sixteen-layer VGG 16 model that had already been trained. The study aimed to find brain tumors (Meningioma, Pituitary, and Glioma). With various processing activities to increase efficiency, the suggested network design detected cancers remarkably effectively. With an accuracy rate of 96%, the CNN model performed substantially better than earlier research.

Manali Gupta et al. (20) used image edge detection and cropping to detect ROI in MRI images, then expanded datasets using data preparation. A basic CNN network containing 14 layers was proposed as a practical classification approach for brain tumors. The approach achieved 96% accuracy, surpassing VGG-16, even with a small dataset, potentially aiding in tumor identification in individuals with brain malignancies.

Zobeda Al-Azzwi1 and Nazarov2 (21) utilized CNN models to classify images of cancer disease using stacking ensemble DL methodology. Three models, VGG19, Inception v3, and Resnet 101, were used to train the data set of unhealthy and normal brains. The 96.6% accuracy demonstrates the efficacy of ensemble models for binary categorization, which was attained by using the Adam optimizer and a Loss binary cross-entropy model.

A novel diagnostic system utilizing CNN with DWT data processing was introduced in (22) to diagnose brain glioma tumors. Discrete Wavelet Transformation (DWT) was used to transfer original MRI pictures to the frequency domain, allowing the suggested CNN model to employ temporal and spatial data instead of typical pixel intensities. The original photographs receive no pre-processing. MRI slices from 382 adult patients are used to train the model. When using the DWT format data and the suggested CNN model, the performance numbers are higher than when using the MRI intensity values as input data. The experimental results demonstrated the superior performance of CNN based on DWT details for binary classification of glioma tumors, with an accuracy of 0.97.

The authors Kibriya et al. (23) suggested developing a 13-layer CNN architecture that is lightweight, has fewer layers, and has learnable parameters to classify brain cancers from MRI data. The proposed model was tested using a benchmark dataset for glioma, pituitary, and meningioma, and it performed best with an accuracy of 97.2%.

Innovative methods for categorizing brain tumor scans were proposed in (24), including hybrid CNNs and transfer learning. There were four classes in each of the 2880 T1-weighted MRI brain scans that made up the dataset (no tumor, glioma, meningioma, and pituitary tumor). The brain tumor types were classified, and two CNNs (AlexNet and GoogleNet) were fine-tuned using transfer learning approaches. They obtained a 97% accuracy rate.

In (25), the study used 3264 MRI images to classify brain tumors using various models. The ResNet-50 model performed best with 80% accuracy, 75% recall, 84% precision, and 75% F1 score. The VGG-19 model had the lowest accuracy rate. The transfer learning approach was more effective than peer studies, especially with small data and fewer epochs. The study highlighted the importance of preprocessing raw MRI scan images for training.

The potential advantages of combining the Python Imaging Library (PIL) with the VGG16 DL algorithm for brain tumor identification have been illustrated by Karamehić and Jukić (26). Accurate and dependable tumor detection has been achieved by integrating the feature collection powers of VGG16 with PIL’s picture preprocessing and processing capabilities. The study’s findings demonstrate the efficacy of this method in accurately classifying various kinds of tumors. With 96.9% accuracy, the research’s methodology delivered reliable identification of tumors across the dataset.

Sarada et al. (27) utilized a modified ResNet50V2 deep learning model to classify four types of brain tumor images. The traditional resNet model was enhanced using batch normalization, maxpooling, and dropout layers. The proposed model achieved an accuracy rate of 96.33%.

Al-Otaibi et al. (28) implemented VGG16 and 2D-CNN to present a unique neural network-based feature engineering technique. Without human involvement, the resulting 2DCNN-VGG16 model extracted spatial features from MRI images. To diagnose brain cancers, machine learning models are then trained using the newly generated hybrid feature set. Using a k-fold accuracy performance score of 0.96.

Shamshad et al. (29). classified benign and malignant brain tumors using MRI images by comparing pre-trained CNN architectures, including VGG16, MobileNet, and ResNet-50. VGG-16 achieves the best accuracy of all the algorithms they propose coming in at 97%.

A unique deep-learning model that makes use of a soft attention mechanism was presented by Mohanty et al. (30) They used a CNN Network with four convolution layers. Their method gathers and combines attributes from all layers instead of obtaining features only from the last layer, as is typical in many models. This guarantees that each layer’s essential qualities are retained and combined into a strong, comprehensive feature vector instead of being lost. The accuracy rate of the suggested model was 95.1%.

Kurniawan et al. (31) used the InceptionResNetV2 structure and data augmentation and Transfer Learning to classify images of brain tumors. In Scenario 1, the accuracy of the suggested architecture used in the test data evaluation was 94.18%. Scenario 2, which paired InceptionResNetV2 augmentation with augmentation, demonstrated an accuracy boost of 95.10%. Moreover, Scenario 3’s accuracy of 96.63% was achieved by combining InceptionResNetV2 via Transfer Learning and augmentation.

This study aims to automate the detection of four types of brain tumors using MRI images. We proposed a classification system using CNN. In the beginning, we used existing activation functions and compared their results. Then, we selected the best-performing function and proposed modifications to enhance its performance further. These modifications improved accuracy compared to the current state-of-the-art.

## Methods

**Figure 1:**
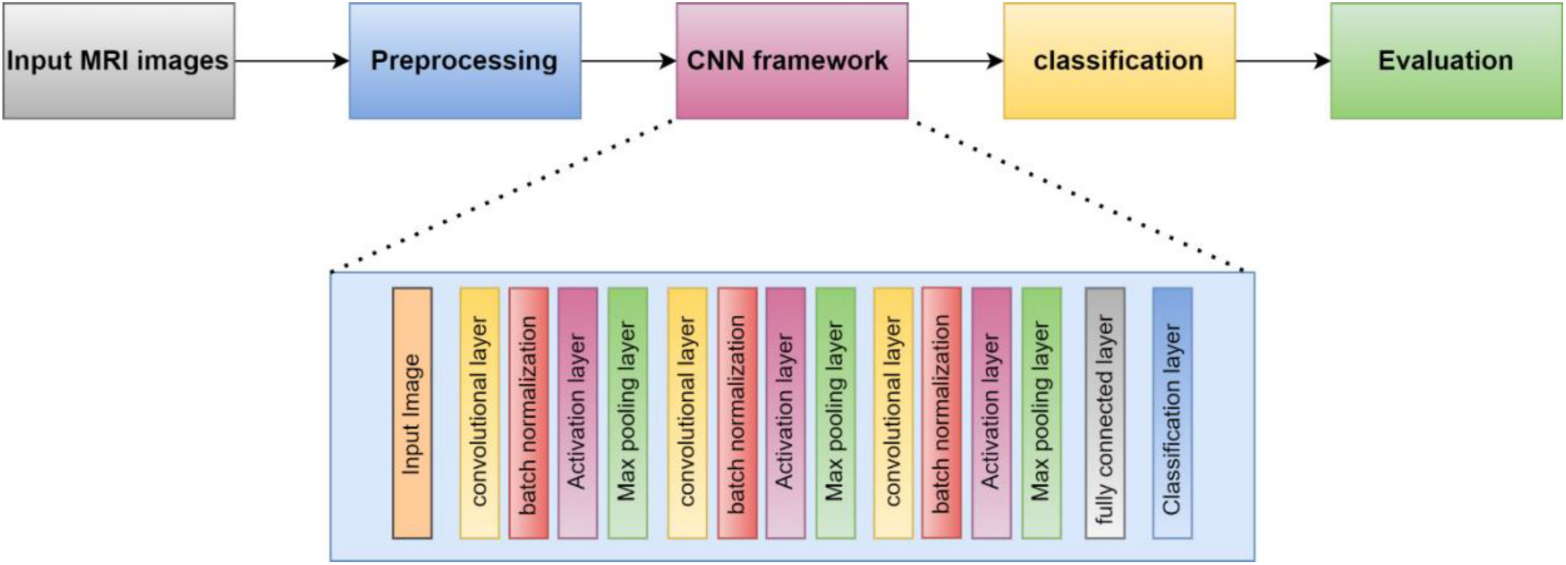
The structure of CNN is proposed.

This section presents the proposed methodology for identifying and categorizing brain cancers using a unique CNN framework. The suggested method has two main parts. First, the dataset must be prepared, and then a custom CNN must be built to extract deep characteristics and classify brain tumors. First, we resized and normalized MRI images. The suggested 16-layered CNN architecture is then fed these images. The general process of the suggested method is depicted in **Figure 1**.

### Dataset

The model was created using the Convolutional Neural Network (CNN) architecture, a popular deep-learning algorithm for image classification. To assess the performance of our model, we used the open-source Brain Tumor MRI Dataset available on Kaggle, a platform for data science competitions and projects, which can be accessed through the following link: https://www.kaggle.com/datasets/masoudnickparvar/brain-tumor-mri-dataset?select=Training. Three datasets (figshare, SARTAJ, and Br35H) were combined to create this dataset (32). The diverse dataset provides the model with a wide range of brain tumor images. This helps the model learn different behaviors and features, improving its ability to predict new, unseen data. As a result, bias towards a specific subset of the dataset is reduced, leading to the development of fairer AI systems. Training on a broad dataset also enhances the model’s robustness and adaptability, allowing it to handle a wider array of situations and data variances.

**Figure 2:**
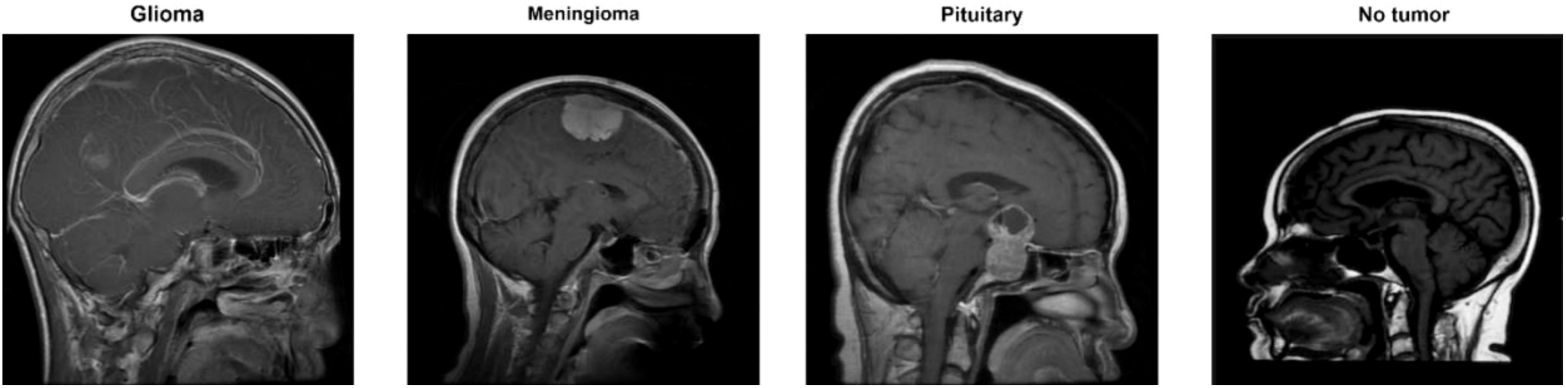
Classes for brain tumors in the dataset (32).

The data collection consists of four classes, as shown in **Figure 2**. These are brain MRI images of healthy people, meningiomas, gliomas, and other tumors. Images for gliomas (1621), meningiomas (1645), pituitaries (1757), and healthy individuals (2000) are available. The figshare dataset includes 3,064 T1-weighted, contrast-enhanced images from 233 patients. These images represent three different types of brain tumors: meningioma (708 slices), glioma (1,426 slices), and pituitary tumor (930 slices). The SARTAJ dataset contains 3,260 T1-weighted, contrast-enhanced images undergoing thorough cleaning and augmentation. Lastly, the Br35H dataset consists of 3,060 Brain MRI Images. **Table 1** presents the information related to the dataset.

**Table 1:** Dataset description.

	figshare	SARTAJ	Br35H	Total
**gliomas**	1426	195		1621
**meningiomas**	708	937		1645
**pituitaries**	930	827		1757
**normal**		500	1500	2000
**Tot**al				7023

There were 7023 MRI pictures used in all. The Kaggle software offers the dataset as open source. The 512 × 512 JPEG images each have a label identifying the kind of brain tumor. Every model used this data set as input data. 70% and 10% of the images were used for the training and validation tasks, respectively. 20% of the images, however, served as test data. However, to give the dataset a suitable input size for every model, resizing was done during the preparation phase.

### CNN

#### Overview

**Figure 3:**
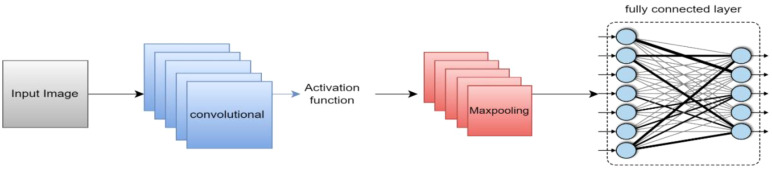
Structure of the suggested approach for classifying brain tumors.

Convolutional neural networks (CNNs) are now the most widely used DL networks. CNNs can process various data inputs, including 1D signals and 2D pictures. An algorithm for deep learning that can assess an input image, rank different visual characteristics according to importance, and differentiate between them is a ConvNet/CNN (33). Compared to other classification methods, ConvNet requires comparatively less preprocessing. While the filters in outdated methods are made by hand, ConvNets can learn about these filters and their attributes. A CNN typically consists of multiple layers, including the input, convolutional, Activation function, fully connected, classification, and output layers. CNN’s core algorithm is a convolution that uses an adaptable filter with preset weight and size values that are changed throughout the training phase’s downsampling to achieve high accuracy. **Figure 3** presents the suggested strategy for this work.

#### Activation function

Neural network activation functions are essential because they compute the weighted total of input and biases to decide whether a neuron can fire or not. Through gradient processing—most commonly gradient descent—they change data and generate an output containing the parameters found in the input. These functions, which can be non-linear or linear, regulate outputs in several fields, such as cancer detection systems, segmentation, object recognition, and speech systems. Here are some commonly used activation functions in deep learning:

i. ReLUThe Rectified Linear Unit (ReLU) is a non-linear activation function that can carry out the derivative operation. While leaving positive values unaltered, the ReLU function makes all negative values zero. The problem of the vanishing gradient can be prevented, which is why ReLU’s simplicity has made it popular (34). It is possible to define the ReLU function mathematically as
(1) $$F\left(x\right)=\;\{\begin{array}{c} \;0,\;x<0\;\\ \;x,\;x\;\geq 0 \end{array}where\;x\;is\;the\;input\;value$$
ii. Leaky ReLUThe “dying ReLU” issue is addressed by the Leaky ReLU function, an adaptation of the ReLU algorithm that allows negative values to descend modestly rather than being set to zero (34).
(2) $$F\left(x\right)=\;\{\begin{array}{c} \;a*x,\;\;x<0\;\\ \;\;x\;,\;\;\;x\;\geq 0 \end{array}where\;a\;is\;the\;scalar\;number$$
iii. Clipped ReLU An update to ReLU called Clipped ReLU applies a threshold operation. Every value entered below zero is set to zero, and any value that exceeds the clipping threshold is set to that limit (36).
(3) $$F\left(x\right)=\;\{\;\begin{array}{l} 0,\;\;\;\;x<0\;\;\;\\ x\;,\;\;\;constant>\;x\;\geq 0\\ constant,\;x\geq constant \end{array}$$
iv. Exponential Linear Units (ELUs)Another kind of AF suggested by Clevert et al. (2015) is called ELUs, which is used to expedite the training of DNN (34). The primary benefit of ELUs is their ability to enhance learning features and mitigate the vanishing gradient issue by employing identity for positive values. Their negative values push the mean unit activation closer to zero, lowering computational cost and accelerating learning (35). Because it reduces bias shifts by driving mean activation towards zero during training, the ELU is a good substitute for the ReLU.
(4) $$F\left(x\right)=\;\{\begin{array}{c} \;\alpha .e^{x}-1,\;\;x<0\;\\ \;\;\;x\;,\;\;\;\;\;\;x\;\geq 0 \end{array}$$
  Where α is the ELU hyperparameter, typically set to 1.0, regulates the saturation level for negative net inputs.
v. Gaussian Error Linear UnitThe Gaussian Error Linear Unit (GELU) was introduced as an alternative to more conventional activation functions like ReLU (37). GELU seeks to offer a smooth and distinct non-linearity to enhance the network’s learning and performance.
(5) $$GELU\left(x\right)=\frac{x}{2}.\left(1+erf\left(0.707*x\right)\right)$$
  In this case, the mathematical function that represents the degree of divergence of a normal distribution over zero is called the error function, or erf(x).
vi. Hyperbolic Tangent FunctionA popular non-linear activation function in neural networks is the hyperbolic tangent function or tanh for short. The conventional tangent function is extended to the hyperbolic space in this way. In some circumstances, the zero-centeredness of the tanh function makes it preferable to the sigmoid function. Tanh, which has a mean value around zero, might enhance learned dynamics in neural networks and lessen the effects of the vanishing gradient issue (38).Tanh function has the following mathematical expression:
(6) $$F\left(x\right)=\;\frac{e^{x}-e^{-x}}{e^{x}+e^{-x}}$$
vii. SoftsignA non-linear AF that transfers the value of the input to a range between -1 and 1 is the Softsign activation function. Apart from the point of origin, the function is continuous, smooth, and differentiable everywhere (36). The following defines the Softsign function:
(7) $$F\left(x\right)=\frac{x}{1+\left|x\right|}$$

The key difference between the tanh and Softsign functions is that the latter converges exponentially, while the former converges in polynomial form.
viii. Swish Activation FunctionIn 2017, Google researchers unveiled the Swish, a non-linear activation function. It is intended to offer a smooth and effective activation function in terms of computing, which may enhance the capabilities of DL (36). The Swish generally possesses a single, uninterrupted positive or negative derivative throughout the construction. Furthermore, it has negative derivatives over some points instead of merely taking positive ones across every point. Through testing on difficult datasets, the developers have shown that the swish function performs better than the widely used ReLU activation function. The mathematical expression for the Swish activation function is:
(8) $$F\left(x\right)=\;\frac{x}{1+e^{-x}}$$
ix. Proposed modified softsign Activation FunctionIn this study, we proposed a new type of activation function by modifying the softsign function. In the positive direction, we kept it as it was in softsign, but in the negative direction, we multiplied it by zero. The mathematical expression for the proposed type is:
(9) $$F\left(x\right)=\left\{\begin{array}{c} \frac{x}{1+\left|x\right|},\;x\geq 0\\ \;0\;,\;\;x<0 \end{array}\right\}$$

The ReLU has a drawback in that it readily overfits when compared to the soft sign function; however, the dropout approach has been used to lessen the effect of ReLU overfitting, and the rectified networks increased the performance of deep neural networks.

**Figure 4:**
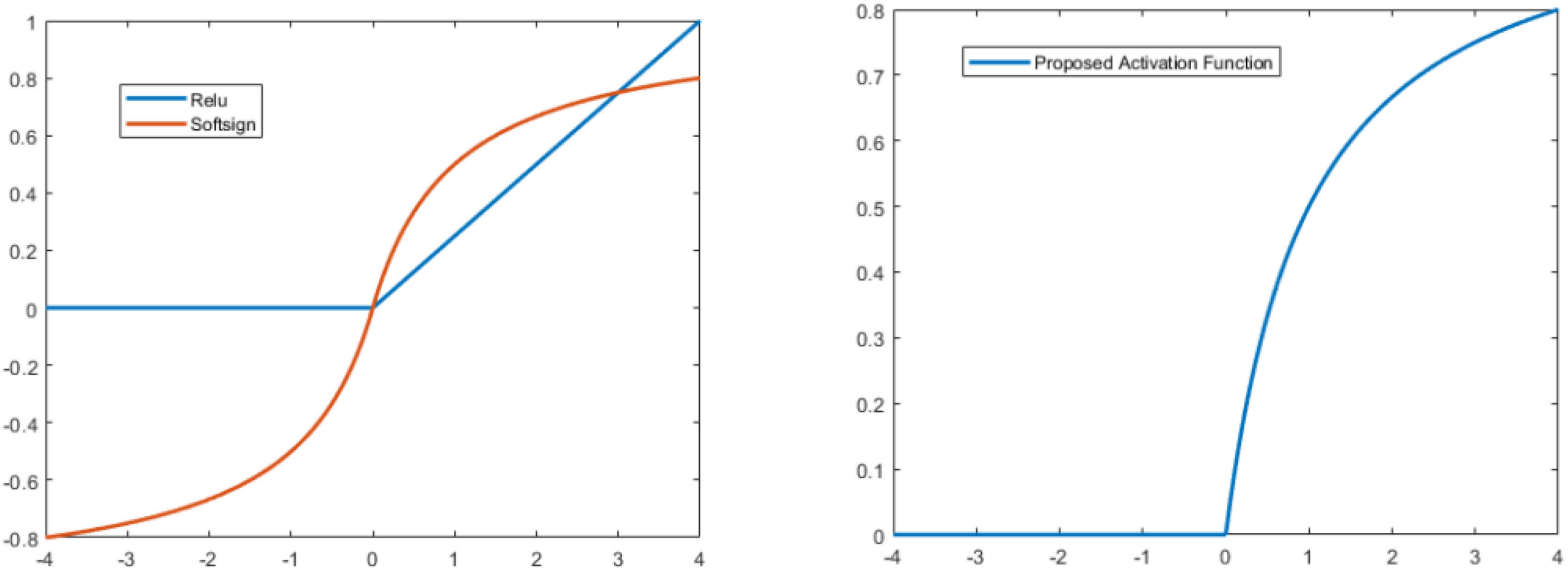
Softsign, ReLU, and proposed functions Comparison Diagram.

Furthermore, the Softsign function increases polynomially rather than exponentially. This softer non-linearity leads to improved and more rapid learning since it eliminates the need to struggle with diminishing gradients. Due to this modification, the learning process is disrupted by dying of some neurons because negative responses cannot discriminate. **Figure 4** compares the Softsign, ReLU, and proposed functions.

#### Proposed CNN

**Figure 5:**
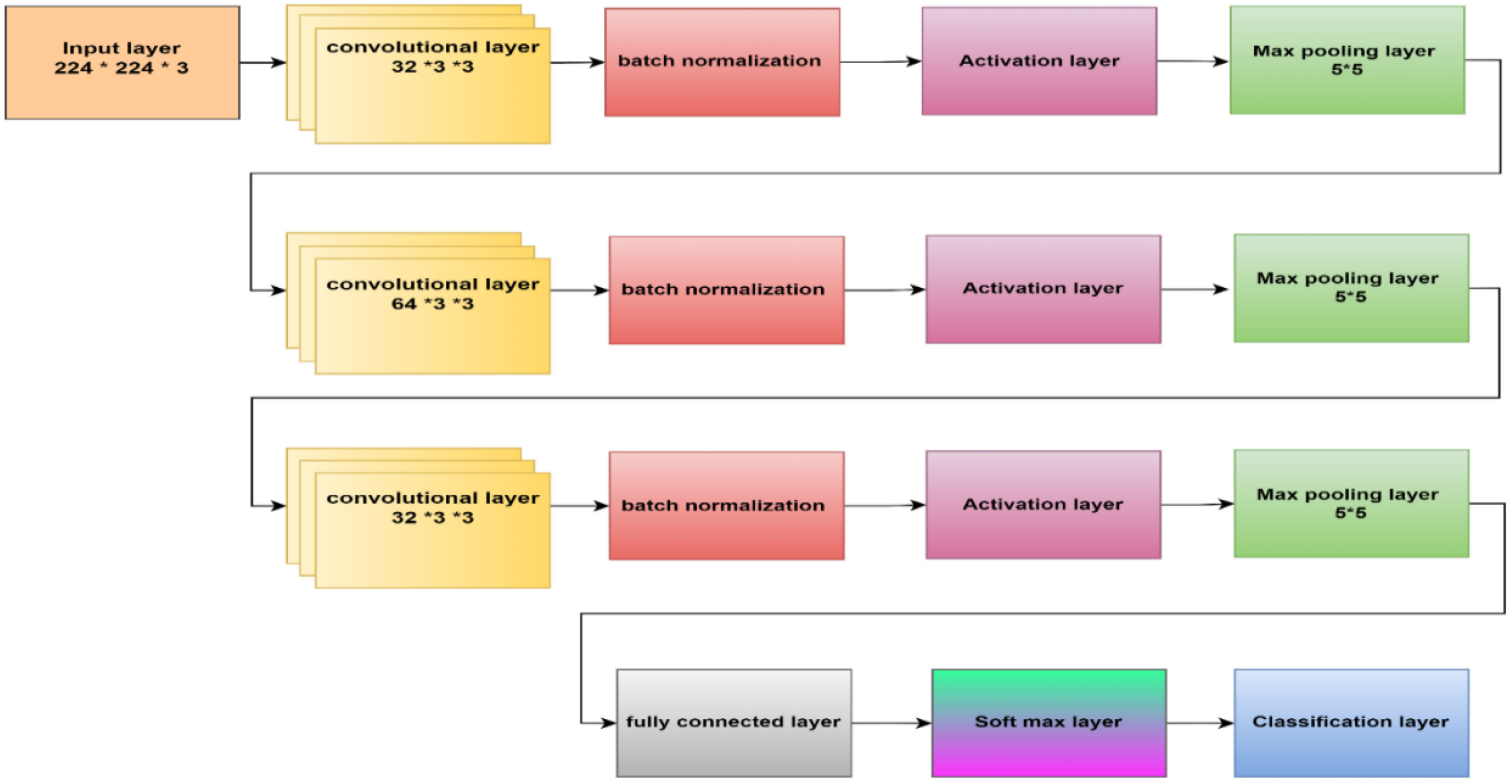
The proposed CNN architecture block.

This paper’s CNN structure started at the input layer, containing an image size of 224 x 224 x 3, as **Figure 5** illustrates. CNN’s structure comprised three convolutional layers to extract deep features and generate the most accurate features map. The batch normalization layer came after each map and oversaw determining the training procedure and lowering the number of epochs needed for CNN structures to learn. The layer with the activation function was placed after the batch normalization layer. The max pooling layer then downsampled the features map. An ultimately linked layer with two neurons was used; in the second stage, this was changed to three neurons.

Within the initial convolutional layer, thirty-two filters with a 3 × 3 size were utilized. Smaller kernel sizes, such as 1x1, 2x2, 3x3, and 4x4, offer advantages over larger sizes like 5x5 and beyond. One of the primary reasons for preferring smaller kernel sizes is their ability to reduce computational costs and promote weight sharing, resulting in fewer weights for backpropagation. However, it is not recommended to use a 1x1 kernel as it generates finely grained and local features without considering information from neighboring pixels. Moreover, the 2x2 and 4x4 sizes are generally less favorable because odd-sized filters symmetrically divide the previous layer’s pixels around the output pixel. Therefore, the optimal choice is a 3x3 kernel. The same kernel size was used for the second and third convolutional layers, which had 64 and 32 filters. To determine the number of filters, we followed a methodology that involved starting with a small number and gradually optimizing them. Our goal was to find the right balance between accuracy and computational efficiency. Ultimately, we selected several filters that allowed us to learn the necessary features effectively. Three × three stride by two padding sizes were used in all convolutional layers. The classification layer was involved to differentiate between the demand classifications. **Table 2** illustrates the configuration of our CNN.

**Table 2:** CNN configuration.

Layer name	Type	Layer configuration	Number of Learnables
imageinput	Input	224 * 224 *3	0
conv_1	Convolution	Number of Filters = 32 Kernel Size =3*3 stride 3*3	896
batchnorm	batch Normalization Layer	scale: 1*1*32	64
functionLayer	activation function		0
maxpool1	Max pooling layer	Kernel Size =3*3 stride 1*1	
conv_2	Convolution	Number of Filters = 64 Kernel Size =3*3 stride 3*3	18496
batchnorm	batch Normalization Layer	scale: 1*1*64	128
functionLayer	activation function		0
maxpool2	Max pooling layer	Kernel Size =3*3 stride 1*1	0
conv_3	Convolution	Number of Filters = 32 Kernel Size =3*3 stride 3*3	18464
batchnorm	batch Normalization Layer	scale: 1*1*64	64
functionLayer	activation function		0
maxpool3	Max pooling layer	Kernel Size =3*3 stride 1*1	0
Fc	fully connected layer	Weights: 4*2592	10372
softmax	soft max Layer		
classoutput	Classification layer		

A thorough study was conducted on the suggested framework, emphasizing accuracy, F1-score, precision, and recall (37). F1-score assesses the trade-off between accuracy and recall, measuring the model’s ability to minimize misclassifying negative instances as positive and recall accurately classifying the appropriate tumor type. Accuracy divides the percentage of correct classifications to determine the model’s overall performance. The receiver operating characteristic curve, or ROC curve, is a graph that illustrates a classification model’s performance overall categorization stages. The term AUC represents “Area under the ROC Curve.” In other words, AUC calculates the area in two dimensions beneath the ROC curve, ranging from (0,0) to (1,1). The AUC score has a value between 0 and 1. The model is better the higher its AUC score.

**Table 3** presents the critical performance metrics alongside their respective mathematical equations.

**Table 3:** Performance metrics.

Name	Definition	equation
Accuracy	Accuracy is defined as the ratio of accurate predictions to the total number of data points.	$\frac{TP+TN}{TP+TN+FP+FN}$
Precision	Precision is the percentage of positive forecasts in the all-positive category.	$\frac{TP}{TP+FP}$
Recall	The amount of true positive (TP) results divided by a total amount of positive class	$\frac{TP}{TP+FN}$
F1-score	F1 score represents a harmonic average of recall and precision.	$2*\frac{\text{Recall}*\text{Precision}}{\text{Recall}+\text{Precision}}$
The receiver operating characteristics (ROC)	The correlation between the rate of true positives and the rate of false positives.	
AUC	the classifier’s capacity to discriminate between classes, which is measured and used to summarize the ROC curve.	

Regarding the previous equations, the number of expected positive instances is known as True Positive (TP). The number of expected negative cases is True Negative (TN). False Negative (FN), often called a (type two) error, is the number of expected adverse events that turn out to be positive. The number of anticipated positive outcomes that are negative is known as a false positive (FP).

## Results and discussion

**Figure 6:**
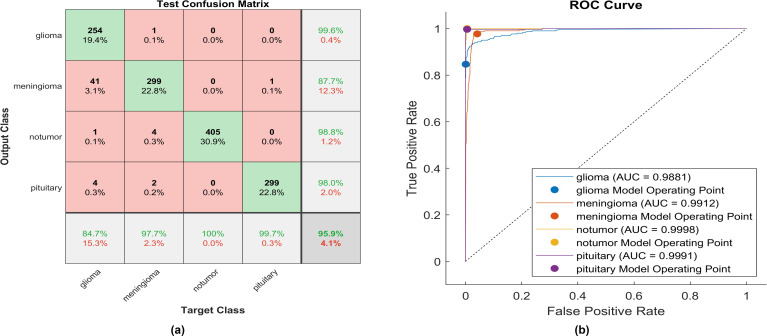
The performance of the ReLU activation layer **(A)** Test confusion matrix. **(B)** ROC curve.

This paper introduces the impact of the activation layer on the classification accuracy for four types of brain tumors. The proposed CNN is trained using various activation layers, as mentioned in the methodology section, and then the trained models are tested. The confusion matrix is generated for each simulation to show how the activation layer affects the accuracy of the brain tumor images. The corresponding confusion matrices are for the testing phase. The data is split into 70% training, 10% validation, and 20% testing. MATLAB 2023a was utilized to carry out the tasks. The simulation was run on an Intel(R) Core i5 CPU with 8GB RAM and Windows 10 software. **Figure 6** shows the performance of the proposed model after using the ReLU activation layer. All experiments are carried out using the following hyperparameters; Adaptive Moment Estimation (Adam) optimizer with initial learning rate of 0.0001, minibatch size of 64, and maximum epoch of 64.

After applying ReLU, 254 out of 300 glioma cases were accurately identified, resulting in a sensitivity of 84.7%. Among these, 41 cases were incorrectly classified as meningioma, one as having no tumor, and four as pituitary. Conversely, one meningioma case was misclassified as glioma. The precision for glioma was high at 99.6%. Furthermore, 299 meningioma cases were correctly identified, yielding a recall of 97.7%. However, four cases were misclassified as having no tumor, two as pituitary, and only one as glioma. Notably, the lowest precision among the four classes was observed for no tumor cases, with a rate of 87.7%. The highest sensitivity was achieved in no tumor cases, reaching 100%, with a precision of 98.8%. Pituitary cases demonstrated promising results in actual predictive rate (99.7%) and precision (98%). The overall accuracy obtained in the experiment is 95.9%. The F1-score, representing the geometric mean between sensitivity and precision, has been evaluated at 95.55%.

**Figure 7:**
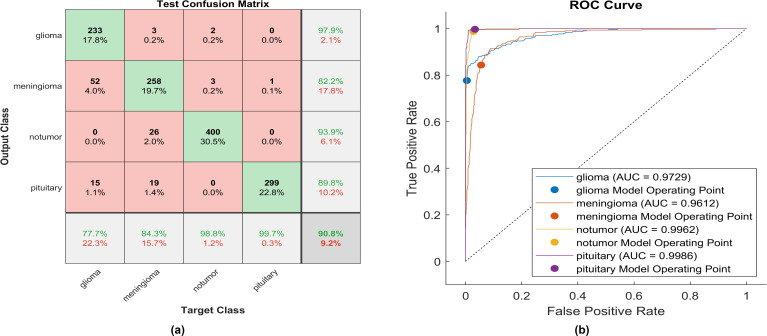
The performance of the Leaky-ReLU activation layer **(A)** Test confusion matrix. **(B)** ROC curve.

The same experiment is carried out using Leaky -ReLU. **Figure 7** shows the performance of the proposed model.

Upon applying Leaky ReLU, 233 glioma cases out of 300 were correctly identified, resulting in a sensitivity of 77.7%. Among these, 52 cases were erroneously classified as meningioma and 15 as pituitary. Conversely, three meningioma cases were misclassified as glioma. The precision for glioma was notably high at 97.9%. Additionally, 258 meningioma cases were accurately identified, producing a recall of 84.3%. However, 22 cases were misclassified as having no tumor, 19 as pituitary, and three as glioma. Notably, a moderate precision among the four classes was observed for no tumor cases, with a rate of 93.3%. The sensitivity in no tumor cases was remarkable, reaching 98.8%, with a precision of 93.9%. Pituitary cases demonstrated the highest results in true predictive rate (99.7%) and a moderate precision of 98%. The overall accuracy achieved using Leaky ReLU was 90.8%. The F1-score, calculated as the geometric mean between sensitivity and precision, is 90.15%.

**Figure 8:**
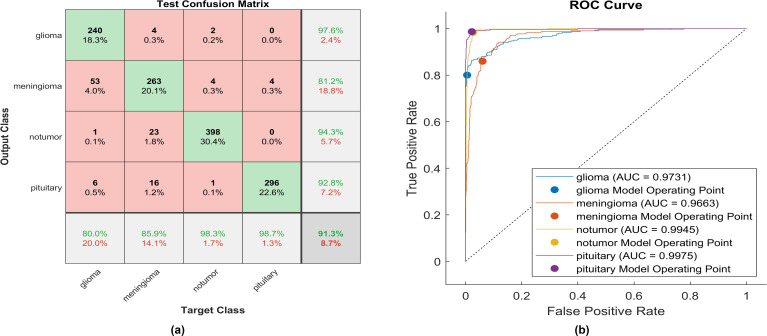
The performance of the clipped ReLU activation layer **(A)** Test confusion matrix **(B)** ROC curve.

An identical experiment was conducted utilizing Clipped ReLU, and **Figure 8** displays the performance of the proposed model.

**Figure 9:**
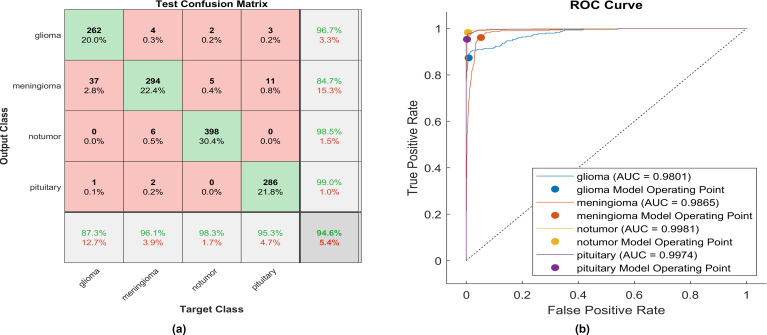
The performance of the ELUs activation layer **(A)** Test confusion matrix. **(B)** ROC curve.

Upon employing clipped ReLU, 240 out of 300 glioma cases were accurately identified, resulting in the lowest recall among all four classes, with a sensitivity of 80.0%. Among these, 53 cases were misclassified as meningioma, one as having no tumor, and six as pituitary. Conversely, four meningioma cases were misclassified as glioma. The precision for glioma was notably high at 97.6%. Furthermore, 263 meningioma cases were correctly identified, yielding the lowest recall of 84.3%. However, 23 cases were misclassified as having no tumor, 16 as pituitary, and four as glioma. Notably, a moderate precision among the four classes was observed for no tumor cases, with a rate of 81.2%. The sensitivity in no tumor cases was remarkable, reaching 98.3%, with a precision of 94.3%. Pituitary cases exhibited the highest results in true predictive rate (98.7%) and a moderate precision of 92.8%. The overall accuracy achieved using clipped ReLU was 91.3%. The F1-score is calculated, representing the geometric mean between sensitivity and precision, resulting in 90.82%. The same experiment uses ELU, and **Figure 9** illustrates the performance of proposed model.

Utilizing ELU, 262 out of 300 glioma cases were correctly identified, resulting in the lowest recall among all four classes, with a sensitivity of 87.3%. Among these, 37 cases were misclassified as meningioma and one as pituitary. Conversely, four meningioma cases were misclassified as glioma. The precision for glioma was notably high at 96.7%.

Additionally, 294 meningioma cases were accurately identified, achieving a high recall of 96.1%. However, six cases were misclassified as having no tumor, two as pituitary, and four as glioma. Notably, the highest precision among the four classes was observed for no tumor cases, with a rate of 98.5%. The sensitivity in no tumor cases was the highest, reaching 98.3%. Pituitary cases demonstrated the highest results in positive predictive value (99.0%) and a moderate recall of 95.3%. The overall accuracy achieved using ELU was 94.6%. The F1-score is assessed, representing the geometric mean between sensitivity and precision, yielding a value of 94.33%.

**Figure 10:**
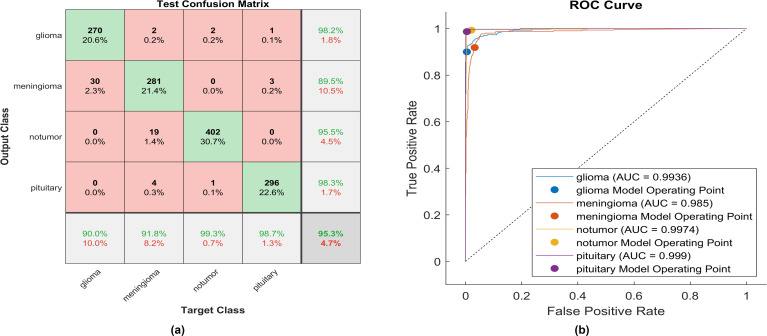
The performance of the GLEU activation layer **(A)** Test confusion matrix. **(B)** ROC curve.

The identical experiment uses the Gaussian Error Linear Unit (GELU), and **Figure 10** depicts the performance of proposed model.

Using GELU, 270 out of 300 glioma cases were accurately identified, resulting in the lowest recall among all four classes with a sensitivity of 90%. Among these, 30 cases were misclassified as meningioma. Conversely, two meningioma cases and two no-tumor cases were incorrectly classified as glioma. The precision for glioma was notably high at 98.2%.

Furthermore, 281 meningioma cases were correctly identified, achieving a high recall of 91.8%. However, nineteen cases were misclassified as having no tumor, four as pituitary, and two as glioma. Notably, the highest sensitivity among the four classes was observed for no tumor cases, with a rate of 99.3%. The precision in no tumor cases was high, reaching 95.5%. Pituitary cases demonstrated the highest results in positive predictive value (98.3%) and a true positive rate of 98.5%. The overall accuracy achieved using GELU was 95.3%. The F1-score is assessed, representing the geometric mean between sensitivity and precision, yielding a value of 95.10%.

**Figure 11:**
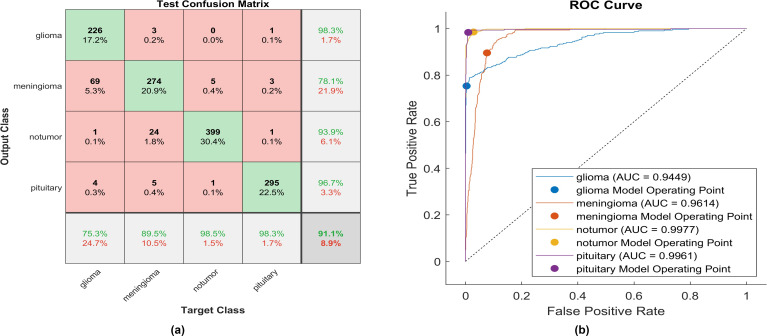
The performance of the Hyperbolic Tangent activation layer **(A)** Test confusion matrix. **(B)** ROC curve.

The activation layer is changed to a Hyperbolic Tangent Function, and the corresponding test confusion matrix depicts the performance of the trained model. An identical experiment was conducted utilizing Hyperbolic Tangent, and **Figure 11** displays the performance of proposed model.

With the utilization of the hyperbolic tangent activation function, 226 out of 300 glioma cases were correctly identified, resulting in the lowest recall among all four classes with a sensitivity of 75.3%. Among these, 69 cases were misclassified as meningioma. Conversely, three meningioma cases and one pituitary case were inaccurately classified as glioma. The precision for glioma was notably high at 98.3%. Additionally, 274 meningioma cases were accurately identified, achieving a high true positive rate of 89.5%. However, twenty-four cases were misclassified as having no tumor, five as pituitary, and three as glioma. Notably, the highest sensitivity among the four classes was observed for no tumor cases, with a rate of 98.5%. The precision in no tumor cases was high, reaching 93.53%. Pituitary cases demonstrated high performance in positive predictive value (96.7%) and a true positive rate of 98.3%. The overall accuracy achieved using the hyperbolic tangent activation function was 91.1%. The F1-score, representing the geometric mean between sensitivity and precision, is evaluated at 90.59%.

**Figure 12:**
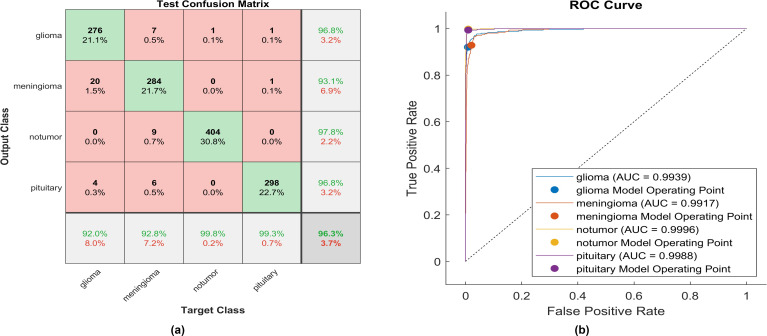
The performance of the soft sign activation layer **(A)** Test confusion matrix. **(B)** ROC curve.

The activation layer has been switched to the soft sign activation function, and the accompanying test confusion matrix illustrates the performance of the trained model. An identical experiment was conducted utilizing soft signs, and **Figure 12** displays the performance of proposed model.

Utilizing the soft sign activation function, 276 out of 300 glioma cases were accurately identified, resulting in the lowest recall among all four classes, with a sensitivity of 92%. Among these, 20 cases were misclassified as meningioma, while four were inaccurately classified as glioma. The precision for glioma was notably high at 96.8%. Furthermore, 284 meningioma cases were correctly identified, achieving a high true positive rate of 97.8%. However, nine cases were misclassified as having no tumor, six as pituitary, and seven as glioma. Notably, the highest sensitivity among the four classes was observed for no tumor cases, with a rate of 99.8%. The precision in no tumor cases was the highest, reaching 97.8%. Pituitary cases demonstrated high performance in positive predictive value (96.8%) and a sensitivity rate of 98.3%. The overall accuracy achieved using the soft sign activation function was 96.3%. The F1-score, which assesses the geometric mean between sensitivity and precision, is determined to be 96.03%.

**Figure 13:**
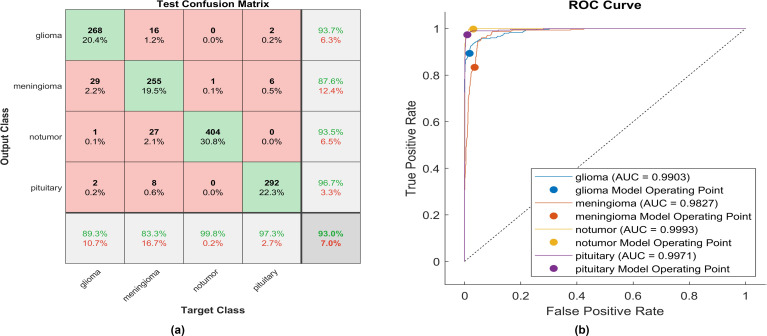
The performance of the swish activation layer **(A)** Test confusion matrix. **(B)** ROC curve.

The activation layer has been changed to the swish activation function, and the corresponding test confusion matrix showcases the performance of the trained model. The same experiment uses swish, and **Figure 13** illustrates the performance of proposed model.

Using the swish activation function, 268 out of 300 glioma cases were correctly identified, resulting in the lowest recall among all four classes with a sensitivity of 89.3%. Among these, 29 cases were misclassified as meningioma, while 16 were inaccurately classified as glioma. The precision for glioma was notably high at 93.7%. Furthermore, 255 meningioma cases were correctly identified, achieving the lowest true positive rate of 83.3%. However, twenty-seven cases were misclassified as having no tumor, and eight as pituitary. Notably, the highest sensitivity among the four classes was observed for no tumor cases, with a rate of 99.8%. The precision in no tumor cases was high, reaching 93.5%. Pituitary cases demonstrated high performance in positive predictive value (96.7%) and a sensitivity rate of 97.3%. The overall accuracy achieved using the swish activation function was 96.3%. The F1-score, representing the geometric mean between sensitivity and precision, is evaluated at 92.61%.

**Figure 14:**
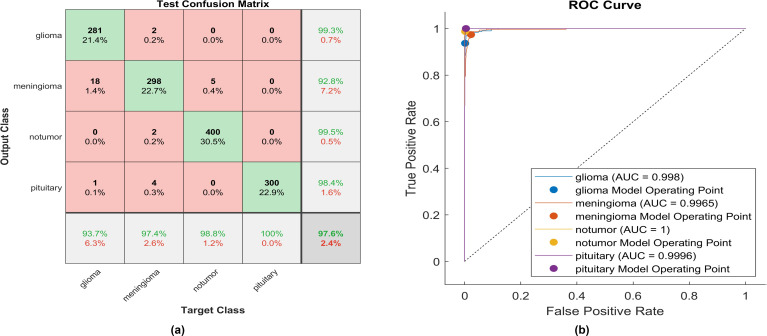
The performance of the proposed activation function is as follows: **(A)** Test confusion matrix and **(B)** ROC curve.

The activation function proposed is a modified version of the soft sign function applied in the activation layer. The model undergoes training and testing, and the performance of the confusion matrix is visually represented in the corresponding figures. **Figure 14A** delineates the instances correctly classified across all classes at the same time; **Figure 14B** illustrates the receiver operating characteristics curve (ROC), which defines the area under the curve (AUC) of the proposed function. This AUC graph depicts the relationship between the true positive rate on the y-axis and the false positive rate on the x-axis.

Applying the proposed activation function, 281 out of 300 glioma cases were accurately identified, resulting in a high true positive rate of 93.7%. Among these, 18 cases were misclassified as meningioma, and only two meningioma cases were inaccurately classified as glioma. The precision for glioma was notably high at 99.3%. Furthermore, 298 meningioma cases were correctly identified, achieving the lowest true positive rate of 92.8%. However, only five cases were misclassified as having no tumor. Notably, a remarkably high sensitivity was obtained in no tumor cases, with a rate of 98.8%. The precision in no tumor cases was the highest, reaching 99.5%. Pituitary cases demonstrated high performance in positive predictive value (98.4%) and the highest sensitivity rate of 100%. The overall accuracy achieved using the proposed activation function was 97.6%. The F1-score, calculated as the geometric mean between sensitivity and precision, is determined to be 97.44%.

Moreover, **Figure 14B** elucidates the superiority of the proposed function with an AUC almost equal to 1 for all classes. This indicates the robustness of the proposed CNN with the proposed activation function in discriminating between the four types of brain tumors. The proposed method overcomes all challenges presented in the previously mentioned activation functions, enabling the utilization of a vast dataset and making the proposed model applicable in healthcare sectors.

The corresponding figure shows the impacts of changing activation functions on the accuracy of the proposed model for classifying brain MR images.

**Figure 15:**
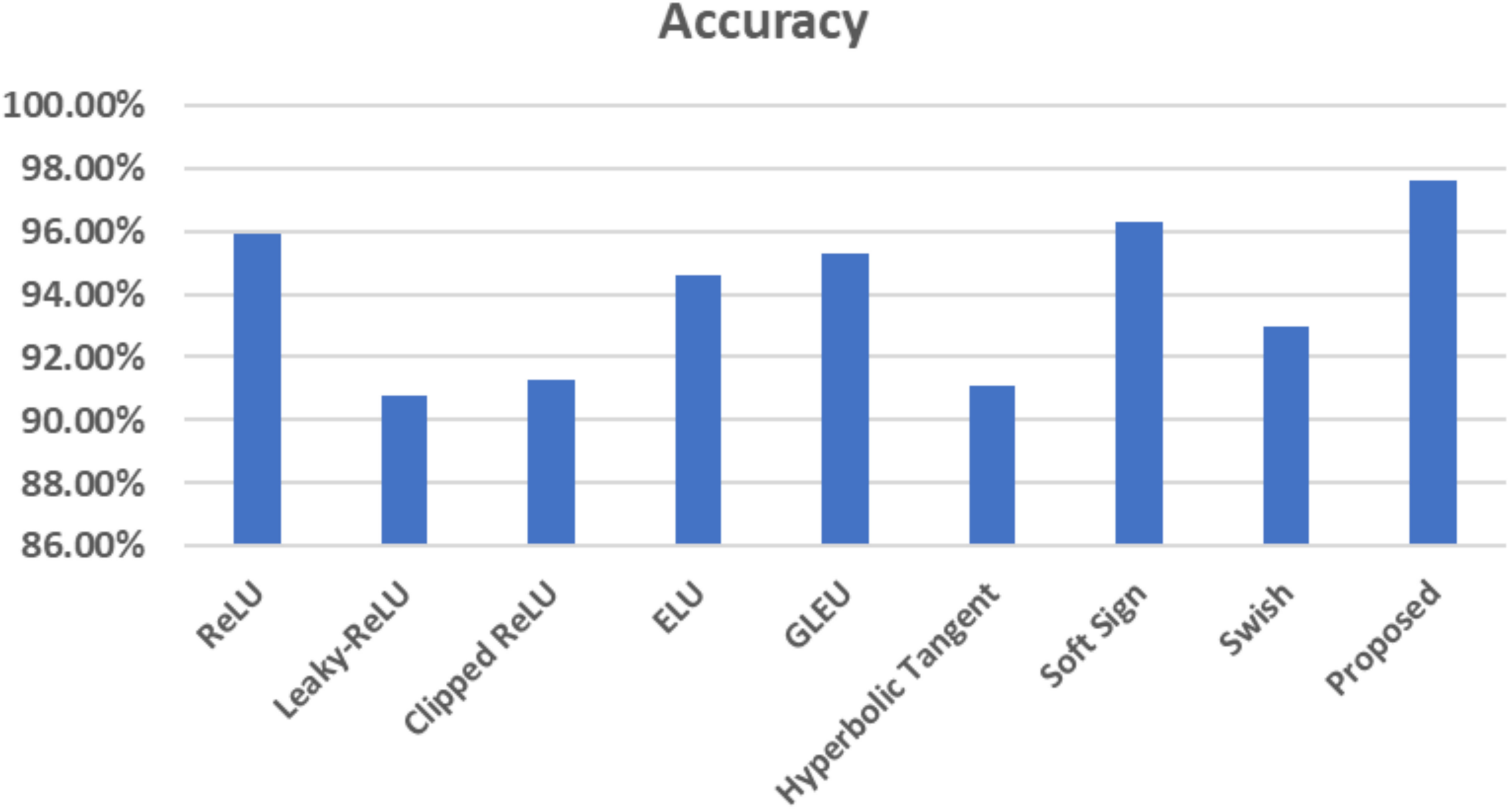
Comparison between various activation functions in terms of accuracy.

**Figure 16:**
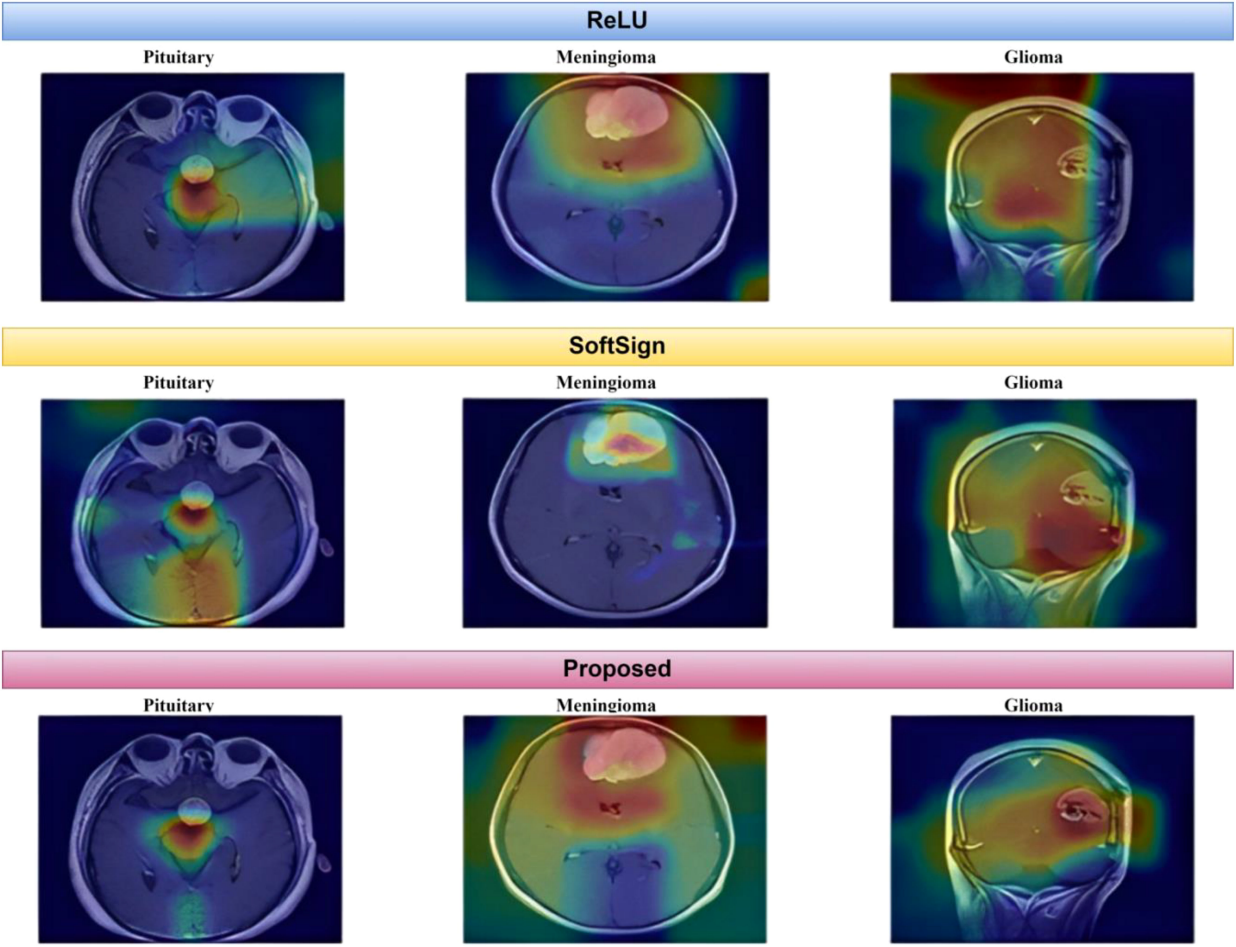
Grad-CAM representations of several tumor types.

As depicted in **Figure 15**, the proposed method exhibits a notable capability in classifying brain MR images into four distinct classes. This approach is characterized by its speed, accuracy, and remarkable results. This paper uses Grad-CAM to generate a class activation map, as shown in **Figure 16**. The degree to which a certain component aids in distinguishing between distinct brain tumors corresponds directly with the color of that component.

A consistent comparative table briefly describes every key feature of every previously published study paper in this field (**Table 4**). **Table 4** is a summary of the strategies used thus far. The collected results, along with some limitations and weaknesses, have been noted for further research.

**Table 4:** Detailed comparison of the results with those from previous studies.

Ref	year	classes	# of Images	Method	Acc	Drawback
14	2023	2	3847	Transfer learning	92.00%	Low accuracy
15	2023	2	696	Transfer learning	96.65%	The employed methodology lacked novelty.
16	2023	3	3064	Transfer learning	91.00%	The employed methodology lacked novelty. Absent a comparative examination.
17	2023	3	3064	Transfer learning	97.00%	Absent a comparative examination. Long Processing time
18	2023	4	3094	Proposed CNN	95.01%	Complex design High rate of learning
1	2023	4	7021	Proposed CNN	96.00%	long time for training
19	2022	3	253	Proposed CNN	96.00%	The small dataset used to train the model
20	2023	2	253	Proposed CNN	96.00%	The small dataset used to train the model
21	2023	2	250	Transfer learning	96.60%	The small dataset used to train the model Absent a comparative examination.
22	2023	2	572	Proposed CNN	97.00%	The small dataset used to train the model Complex design
23	2022	3	3046	Proposed CNN	97.20%	
24	2023	4	2880	Transfer learning	97.00%	Basic Model
25	2022	4	3264	Transfer learning	80.00%	The employed methodology lacked novelty. Low accuracy
26	2023	4	7021	Transfer learning	96.90%	The employed methodology lacked novelty. Basic Model
27	2024	4	7023	Modified ResNet50V2	96.33%	long time for training
28	2024	4	21,672	Transfer learning	96.00%	Complex design
29	2024	4	3096	Transfer learning	97.00%	The employed methodology lacked novelty.
30	2024	3	11,538	Proposed CNN	95.10%	long time for training
31	2024	4	3264	Transfer learning	96.63%	The employed methodology lacked novelty.
	**The proposed Method**	**4**		**Novel CNN with Novel activation function**	**97.6%**	**New approach with new Activation function**

## Conclusion

Convolutional neural networks for brain tumor classification have paved the way for improved tumor identification and accuracy. MRI is the most extensively used method for detecting and classifying brain tumors. Because of their apparent efficient feature extraction capabilities, DL-based algorithms have recently received increased attention and efficiency compared to traditional medical imaging classification techniques. If cancer is detected, many lives can be saved, and the appropriate grade is identified using simple and inexpensive diagnostic methods.

This work provides a new CNN-based approach for MRI image classification. The four classes into which the proposed approach can identify tumors are Glioma, Meningioma, Pituitary, and No-tumor. In deep learning designs, activation functions determine whether information should be transferred to the next neuron. There are 16 layers in total in the architecture, including layers for convolution, batch normalization, activation function, and maxpooling. In this work, we experimented with several types of activation functions, and in addition, we proposed a new type of activation function.

Our suggested approach achieved 97.6% accuracy on 7023 MRI pictures from the dataset, which is accessible to the public. The proposed CNN architecture does not require manual lesion segmentation before classification. The results show that our proposed approach is more effective than current approaches and that physicians can use it to identify and categorize tumor types from MRI scans in real-time. Even though the performance of our suggested approach is promising, in future work, we will use a variety of imaging modalities and segmentation approaches to obtain the best estimation of affected areas in the brain and isolate them from unaffected regions. Different modalities with image registration differences will provide essential image traits in the fixed image and perform the best classification, improving precision and accuracy.

In addition, we plan to expand our database and analyze a larger set of data in the future to design and conduct clinical experiments more efficiently, deepening our understanding of brain cancer behavior and therapeutic responses from different groups of patients.

## Data Availability

The original contributions presented in the study are included in the article/supplementary material. Further inquiries can be directed to the corresponding author/s.
